# Analysis of Spatial Heterogeneity of Responses in Metastatic Sites in Renal Cell Carcinoma Patients Treated with Nivolumab

**DOI:** 10.3390/tomography8030110

**Published:** 2022-05-20

**Authors:** Ankush Jajodia, Varun Goel, Nivedita Patnaik, Sunil Pasricha, Gurudutt Gupta, Ullas Batra, Vineet Talwar

**Affiliations:** 1 Department of Radiology, Juravinski Hospital, Hamilton Health Sciences, McMaster University, Hamilton, ON L8S 4L8, Canada; 2 Department of Medical Oncology, Rajiv Gandhi Cancer Institute and Research Centre (RGCIRC), New Delhi 110085, India; ullasbatra@gmail.com (U.B.); talwar.vineet@rgcirc.org (V.T.); 3 Department of Histopathology, Rajiv Gandhi Cancer Institute and Research Centre (RGCIRC), New Delhi 110085, India; patnaik.nivedita@rgcirc.org (N.P.); drsunilpasricha@yahoo.com (S.P.); drggupta@yahoo.com (G.G.)

**Keywords:** renal cell carcinoma, organ-specific response, Nivolumab

## Abstract

**Background:** The purpose was to determine whether tumor response to CPI varies by organ and to characterize response patterns in a group of surgically treated metastatic RCC patients treated with Nivolumab. **Methods**: A retrospective analysis was undertaken between January 2016 and March 2020 on patients receiving Nivolumab for metastatic RCC, following first-line therapy and having at least one baseline and two follow-up scans. A Fisher’s exact test was used to compare categorical variables, and a Kruskal–Wallis test was used to compare continuous variables. **Results:** Twenty-one out of thirty patients evaluated were eligible, and they were divided into two groups: responders (n = 11) and non-responders (n = 10). According to all iRECIST standards, 18 (85.7 percent) of the 21 patients had PD (10 patients), PR (3 patients), or SD (8 patients). At baseline, 7, 15, 4, 13, 7, and 7 patients, respectively, had detectable hepatic metastasis and lung, brain, lymph node, soft tissue, and other intra-abdominal metastases; these patients were evaluated for organ-specific response. The ORRs for hepatic metastasis and lung, brain, lymph node, soft tissue, adrenals, and other intraperitoneal metastases were correspondingly 10%, 20%, 35%, 0%, and 25%. In total, 13 (61.9%) of them demonstrated varied responses to CPI therapy, with 6 (28.5%) demonstrating intra-organ differential responses. The lymph nodes (35%) had the best objective response (BOR), followed by the adrenals and peritoneum (both 25%), the brain (20%), and the lung (20%). The response rate was highest in adrenal gland lesions (2/4; 50%), followed by lymph nodes (13/19; 68.4 percent) and liver (5/10; 50%), whereas rates were lowest for lesions in the lung (9/25; 36%), intraperitoneal metastases (1/4; 25%), and brain (1/5; 20%). **Conclusions**: In renal cell carcinoma, checkpoint inhibitors have a variable response at different metastatic sites, with the best response occurring in lymph nodes and the least occurring in soft tissue.

## 1. Introduction

Renal cell carcinoma (RCC) accounts for roughly 3% of all cancer cases [1], with a nefarious presentation of distant metastatic lesions at the time of diagnosis in 25% to 30% of patients, associated with significant mortality [2]. Despite curative surgery such as radical nephrectomy, around 30% of patients develop metastases [3]. Targeted treatments against VEGF and mammalian target of rapamycin inhibitors have significant toxicity due to the pharmacological modes of action [4,5,6]. Immunotherapies have shown long-term effects, improved overall survival (OS), and tolerability [7,8,9,10]. Nivolumab is a fully human IgG4 PD-1 immune checkpoint inhibitor (CPI) antibody that inhibits the interaction of PD-1 with its ligands PDL1 [8]. Clinical trials (phase 3 Checkmate 025 study) have shown that utilizing nivolumab improves overall survival in patients with advanced renal cell carcinoma who had previously been treated with everolimus [8].

New patterns of innovative treatment responses have arisen due to the introduction of these new immune treatments. Heterogeneous responses to CPI treatments have been postulated due to the combined action of diverse mechanisms of action, molecular targeted therapy, and the interplay of the varied tumor microenvironments of various organs. Several studies have found that patients with melanoma have mixed responses to immunotherapy: metastatic tumors in one organ expand, whereas those in another improve or remain stable [11,12,13]. One of the several distinctions in assessing tumor burden response to immune therapy vs. traditional cytotoxic medications is the long lag time for adequate response, which necessitates considering a durable, stable disease (SD) to assess antitumor efficacy. Another inimitable non-conventional response allied with immune therapy is pseudoprogression, which is histologically defined as tumor development until either a sufficient immune response improves, or transitory immune cell infiltrates [14,15]. Version 1.1 of the Response Evaluation Criteria in Solid Tumors (RECIST) is insufficient for capturing Pseudo progression (PsPD) and underestimating immune checkpoint blockade’s therapeutic efficacy. The irRECIST (immune-related) [16] simplifies existing immune-related response criteria (irRC) [17] to quantify tumor response better, converting the two-dimensional assessment to a one-dimensional measurement, and was used in clinical trials [16]. The concepts of objective tumor response for the most recently introduced iRECIST remain intact from RECIST 1.1. One noteworthy notion isg that the bar for progression is reset if tumor progression at the current time point is followed by response or stable disease follow-up scans [18].

This study sought to determine if tumor response to CPI in RCC varies by organ and to describe response patterns and discrepancies across RECIST 1.1 and iRECIST criteria assessments in a group of surgically treated metastatic RCC patients receiving Nivolumab.

## 2. Materials

### 2.1. Patient Population

This retrospective research ethics committee-approved study, conducted at a single center, aims to ascertain the patterns of response and recurrence to nivolumab (Opdivo^®^, Bristol-Myers Squibb Company, New York, NY, USA). We evaluated patients presenting with metastatic RCC in the medical oncology care unit of RGCIRC, who had received at least one nivolumab infusion with the standard of care, 3 mg/kg every two weeks, between May 2017 and January 2020; all patients were treated at the Rajiv Gandhi Cancer Institute and Research Centre. Patients were enrolled only if they had measurable diseases and subsequent imaging examinations were accessible for response evaluation. The electronic patient record system was used to extract clinical data.

### 2.2. Imaging Examination

All CT examinations were performed on a Siemens Healthineers dual-source CT system (Forchheim, Germany). The following scanning parameters were used: tube voltage 120 kV, automated tube current modulation (30–70 mAs), pitch 1.0–1.5 mm, matrix 512 × 512, slice thickness 5 mm, the field of view 350 × 350 mm, slice thickness of 0.625–1.25 mm. All measurements were made using an electronic calipers tool on the axial image data by experienced radiologists, in up to five lesions in total and up to two lesions per organ for target lesions. CTs were scheduled every three months until RECIST 1.1-defined progressive illness (PD), death, or patient refusal, whichever occurred first.

All study cases were evaluated using documented and validated RECIST 1.1 and Irecist [18,19]. In subsequent iRECIST assessments, the target lesion measures obtained using RECIST 1.1 were employed for each time point. At each time point, the overall response, the best overall response (BOR), and the time point at which worsening disease was noted were reported in RECIST 1.1. For iRECIST, data were collected at each time point on the target lesion response and the non-target lesion response, new lesion response, and overall response.

Serial computed tomography (CT) scans were assessed for overall response rate (ORR) and organ-specific response rate (OSRR) using the response evaluation criteria in solid tumours (RECIST) version 1.1 [20]. OSRR evaluated a maximum of five target lesions per organ. Non-target lesions (all other lesions, including problematic lymph nodes) were tracked as present, absent, or progressing unequivocally. Lymph nodes were categorized as a single organ regardless of their location. Because lymph nodes were classified as organs, the longest diameter of the lesion was assessed similarly to other organ locations as defined by RECIST 1.1.

### 2.3. Analytical Statistics

All statistical analyses were executed in software packages R version 3.4 (The R Foundation for Statistical Computing). Fisher’s exact test was used for categorical variables and the Kruskal–Wallis test for continuous variables. TTP was calculated using Kaplan-Meier. All p values are two-sided. A *p*-value of 0.05 or less was deemed significant.

## 3. Results

### 3.1. Patient Characteristics and Clinical Outcomes

There were 21 eligible individuals among the 30 mRCC patients in this retrospective study. The patients were divided into responders (n = 11) and non-responders (n = 10). The median age was 58 years (range 33–70). The average length of time in therapy was 2–31 months (median = 14 months). Table 1 summarizes the clinicodemographic characteristics as well as the histological parameters. According to iRECIST guidelines, ten patients had PD, three patients were PR, and eight had SD. While on CPI medication, eight patients experienced clinical problems, amounting to death in three (cardiac event = 1, immune-confirmed progressive illness = 2). Other complications encountered during the study period included pneumonitis (n = 1) due to CPI therapy, IVC thrombus (n = 1), (intra-abdominal metastasis invading the IVC), deranged liver function test (n = 1), duodenal hemorrhage (n = 1, treated with interventional angioembolization of the gastroduodenal artery), and colonic perforation (n = 1, treated with surgical laparotomy exploration and hemicolectomy). Both patients were stable after the intervention till the end of the research study. Eight patients (38%) exhibited early progression (labeled IUPD according to iRECIST criteria at the initial CT examination), confirmed as ICPD in six individuals by a repeat CT 4–8 weeks later or by death due to tumor progression.

### 3.2. Organ-Specific Responses

**Figure 1:**
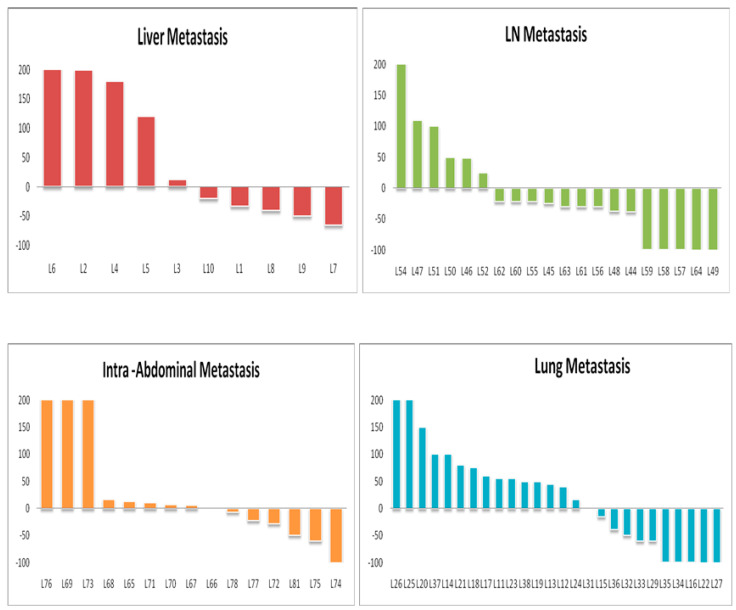
Best percentage change over time (from baseline) in tumor burden in various organ systems.

**Figure 2:**
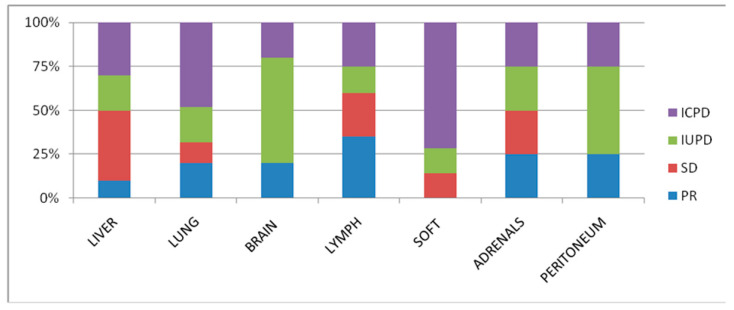
Organ-specific response in the liver, lung, brain lymph node, soft tissue, adrenals, and peritoneal metastases.

The objective response rate (ORR) according to RECIST 1.1 was 35% and 50% as per iRECIST. Overall, at baseline, 7, 15, 4, 13, 7 and 7 patients had measurable hepatic metastasis and lung, brain, lymph node, soft tissue, and peritoneal metastases, respectively; these patients were subject to organ-specific response evaluation. Organ-specific response rates (OSRR) of hepatic, lung, brain, lymph node, soft tissue, adrenals, and intraperitoneal metastases were 10, 19, 20, 35, 0, 25, and 25%, respectively (Table 2). The best percentage change in tumor burden relative to that at baseline in different organ systems is represented in Figure 1 and Figure 2. The best objective response (BOR) was seen in lymph nodes (35%), followed by adrenals and peritoneal (25% both), and worst in the liver and metastatic soft tissue lesions (10 and 0%). Thirteen patients (61.9%) exhibited differential responses to CPI treatment, with six (28.5%) patients revealing intra-organ differential responses.

### 3.3. Comparison of the RECIST 1.1 and iRECIST Criteria at the First Occurrence of Progression

Among the 76 lesions, there were 34 (44.7%) discordant assessments between the iRECIST at the first and second follow-up; this might have a theoretical influence on the therapeutic decision. Ultimately, four patients (19%) had the clinical benefit, initially characterized as PD by the RECIST1.1 criteria in final follow-up imaging, but had treatment benefit following iRECIST. Of the 14 patients with confirmed PD of target lesions confirmed by iRECIST, 5 (35.7%) had an atypical response on imaging (all dissociated responses) not recognized by the criteria according to the definition of the initial target lesions.

### 3.4. Response Assessment by iRECIST and RECIST1.1

**Figure 3:**
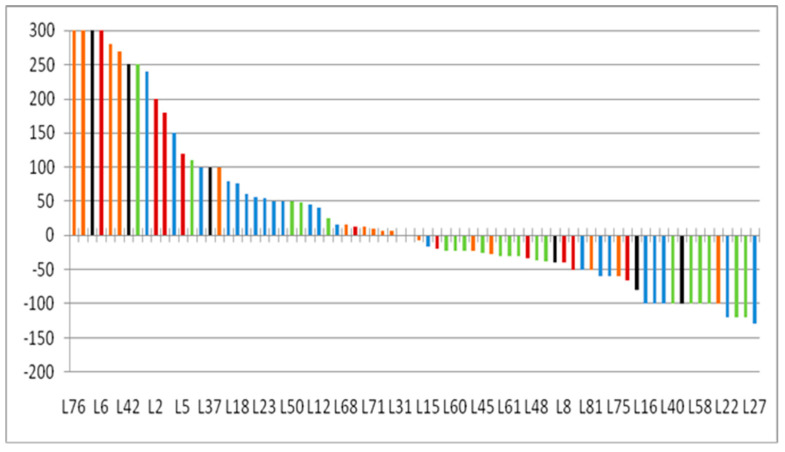
Waterfall plot of tumor burden change at the best overall response in all lesions.

**Figure 4:**
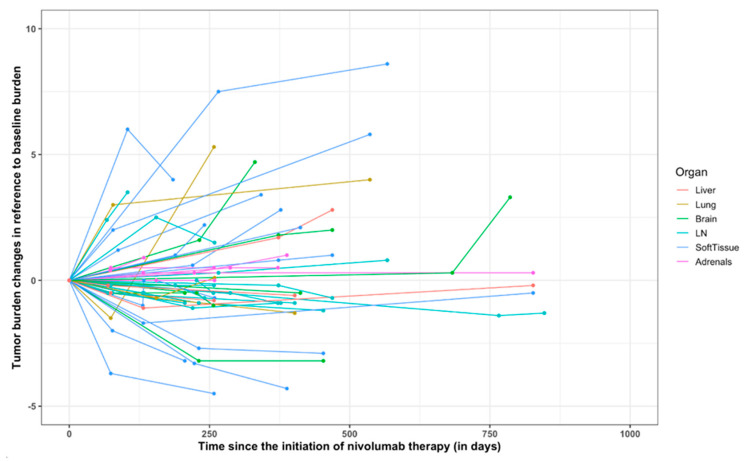
Spider plot of tumor burden changes during nivolumab therapy using irRECIST. Longitudinal changes of tumor burden during therapy are shown about baseline, showing baseline tumor burden as 0.

Tumor burden changes about baseline (%) at the time point of best overall response in 21 patients ranged from −64 to +185% (median: −12%) (Figure 3). Discordance of BOR was noted in 4 (19%) patients, in which BOR was iSD by iRECIST1.1 but was PD by RECIST1.1. The remaining 3 patients had IUPD at the endpoint of the study as defined by iRECIST but were confirmed progress by RECIST1.1. The tumor burden changes during nivolumab are demonstrated in the spider plot (Figure 4). None of the patients in this study confirmed pseudoprogression.

### 3.5. New Lesions during Nivolumab Therapy

Eight patients (8/21; 38%) developed new lesions during therapy. Two patients developed new target and non-target new lesions, and six developed new target lesions alone [range 1–3 per patient]. The most common location of new target lesions was the liver (n = 3), followed by lymph nodes and brain (n = 2), adrenal gland (n = 1), lung, and peritoneal metastasis (n = 1). According to iRECIST, these eight patients were qualified as iSD (n = 3), iPD (n = 4) and IUPD (n = 1).

### 3.6. Lesion-Based Response Assessment

**Figure 5:**
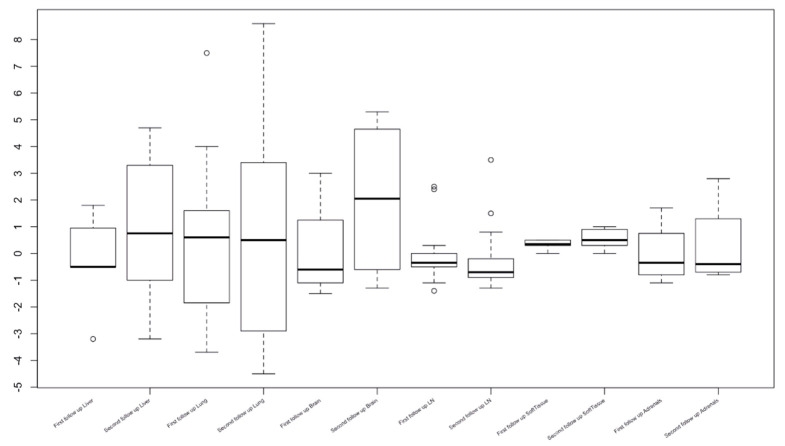
Lesion-based tumor size change at the best response of 76 target lesions classified by the organs. The black horizontal line shows a median value for the lesion-based shrinkage in each organ. The gray vertical line with horizontal bars at the upper and lower ends represents the first and third quartiles.

Nineteen patients (90.4%) had more than one lesion in the same organ. Lesion-based tumor size change (%) at the best response of each lesion was significantly different across the organ categories (Kruskal–Wallis *p* = 0.003). Adrenal lesions and lymph nodes had more significant shrinkage, followed by lung, whereas liver and miscellaneous lesions had less shrinkage (Figure 5). According to the location, the response rates also significantly differed among the lesion groups (Fisher *p* = 0.02).

## 4. Discussion

A direct comparison of iRECIST and RECIST1.1 evaluation outcomes for mRCC patients treated with nivolumab in the clinical context is presented in this study, which reveals the early results of tumor response characteristics using iRECIST. During the research, no patient showed signs of pseudoprogression. Adrenal and lymph node lesions responded better to therapy than liver lesions based on a lesion-based evaluation, which revealed substantial disparities in responses across organs. Additionally, our findings corroborate preceding reports demonstrating decreased activity in liver metastases [21] when the liver possesses inhibitory immunomodulatory properties [22]. However, this observation remains to be confirmed in a larger patient population due to the small number of patients with liver metastases included in our analysis. The liver has an immune suppressive microenvironment that may help tumors escape antitumor immune attacks during therapy, resulting in less tumor shrinkage. The adrenal gland is believed to have immunomodulatory capabilities [22], and in the Nishino et al. [23], the adrenals demonstrated a high response rate in the lesion-based assessment. In congruence with these findings, our cohort’s OSRR levels were high in the adrenals, though the small number of individuals with adrenal metastases precluded a decisive declaration. The adrenal gland is a significant organ of the hypothalamic-pituitary-adrenal (HPA) axis, responsible for cytokine synthesis and action. As a result, the organ is known to have immune-modulatory properties via activation of the HPA axis and cell-cell mediated immune-adrenal interactions. Additional research is needed to elucidate nivolumab’s effect in adrenal metastases compared to other sites, as another study showed a low response rate in adrenals with metastatic non-small cell lung cancer [22].

Immune checkpoint inhibitor response may also be influenced by the tumor microenvironment (TME), which consists of a variety of cell types, including fibroblasts, endothelial and immune cells. This could potentially impaire nivolumab’s activity, which is dependent on the location of metastases, which typically have a different proportion of immune cells under physiologic state [24]. Recent research indicates that lymphocytes invading tumors within lymph node metastasis is connected with a more favorable prognosis. Nishino et al. [23] observed high OSRR in lymph node metastases, corroborating the hypothesis of improved response in organs with high pre-treatment immune cell infiltration. OSRR in lung metastases and original tumors would be high due to the immunologically active microenvironment.

Additionally, the liver is thought to be an organ that inhibits immunological modulation, which can suppress immunological responses and cause immunity tolerance [25]. This finding is corroborated in our study, as patients with LN metastasis fared well relative to those with soft tissue and liver metastases. This finding also resonated in previous clinical trials involving patients with melanoma or non-small cell lung cancer (NSCLC); decreased response rates and shorter progression-free survival were seen in patients with melanoma or NSCLC who had liver metastases, compared with those who did not have liver metastases [21]. A variable immune cell infiltration before therapy may result in variable nivolumab action based on the organ in question, hence justifying the investigation of organ-specific radiologic response rates to nivolumab (OSRR). The distribution of nivolumab in various organs is clinically significant in assisting in radiological surveillance during therapy and may identify people with oligoprogressive illnesses who are amenable to oligoprogression to local therapy that is additive.

The discordance in BOR evaluation between two criteria was detected exclusively between SD and PD, either due to the confirmation need for PD or irRECIST1.1’s inclusion of new lesions in the total tumor burden, which contributed equally to the BOR discordance. Pseudoprogression, defined as early progression characterized by an increase in tumor burden followed by a subsequent response, is a complicated phenomenon to treatment with immune checkpoint inhibitors. None of the patients in this study experienced pseudoprogression during therapy, which may be explained by the small number of patients studied in such a short period during this initial clinical experience. Following immune-checkpoint inhibitor therapy, the development of new lesions adds another layer of complexity to management since it can signal genuine progression or pseudoprogression. One-third (38%) of patients in the current study had new lesions, most of which were in the liver. The majority of patients who underwent a follow-up scan following the formation of new lesions demonstrated progression of new lesions, with none of the new lesions responding to therapy. A recent study [26] evaluated tumor responses in various metastatic and primary sites in patients with RCC, who underwent nivolumab monotherapy, with site-specific overall response rates (ORRs) as: lung (36%), bone (5%), lymph node (33%), liver (50%), adrenal gland (29%), pancreas (33%), and brain (0%). Interestingly, our study reports comparable values, except we encountered 10% response rates in the liver, which was significantly less than this study. This may be due to differences in the number of subjects and measurement criteria.

A salient finding in our study was the excellent response observed in a population with sarcomatous elements (28%). TME may have a role in the observed response disparities between sarcomatoid dedifferentiated tumors. Subgroup analyses of sarcomatoid patients treated with combination CPI or VEGF plus CPI therapy revealed impressive response rates [27]. The increased expression of PDL1 and T-effector gene signatures in these tumors relative to their non-sarcomatoid counterparts may justify the robust responses to combination CPI therapy. The results of a biomarker-driven, open-label, non-comparative, randomised phase 2 trial [28] show that a prospective patient selection based on the molecular phenotype of the tumor is possible, and has a positive effect on choosing the most effective treatment (between nivolumab, with or without ipilimumab, and a VEGFR-TKI) in the first-line treatment of metastatic clear-cell renal cell carcinoma. It is not feasible in our study to assess the molecular group determination in primary or metastatic site in all patients; however, this is a potential area of interest for future studies.

Our study was not without limits. To begin, our study was retrospective, with a small sample size of patients with renal and extrarenal malignancies. Our findings require confirmation in prospective, large-scale research. Second, we did not use modified RECIST (mRECIST) to assess tumor responses, which means the ORR may be underestimated. However, we believe that mRECIST is more appropriate for evaluating patients who have undergone antiangiogenic therapy, and the majority of current clinical trials testing CPI use RECIST 1.1 or iRECIST irrespective of tumor location [29]. Third, the patients in our study had a range of prior systemic therapy and tumor evaluation frequency. It is unknown if these factors influence organ-specific differential responses to CPI and can only be addressed in more homogeneous cohort research. Fourth, our study did not investigate the processes behind organ-specific differential responses to CPI, as this would require collecting tumor samples from different organs of the same individual, simultaneously. Finally, we did not evaluate the differences in progression-free survival using the different response assessment criteria; however, this has been voiced in previous large-scale clinical trials, which were beyond the scope of this study [30].

## 5. Conclusions

In conclusion, our study shows that individuals with advanced mRCC had organ-specific variable responses to ICIs. Further investigation of the underlying process is warranted. Our findings support the concept that immunotherapy efficacy is location-dependent. Treatment appears to work better in lymph nodes and adrenals than in the soft tissue metastasis and liver. Additional local treatment may be used in the future if oligoprogression occurs in these organs despite prolonged therapeutic improvement.

## Figures and Tables

**Table 1:** Baseline patient characteristics.

Clinical Parameters	Total N = 21 (%)	Responders (N = 11)	Non-Responders (N = 10)
**Age**	33–70 (Median = 58 yrs)	33–70 (Median = 51 yrs)	46–69 (Median = 58 yrs)
**Sex (M/F)**	20/1 (95.2%/4.8%)	10/1 (90.9%/9.1%)	10/0 (100%/0%)
**Smoking**	4 (19%)	3 (27.2%)	1 (10%)
**Co-morbidity** (Diabetes, Hypertension and COPD)	12 (57.1%)	7 (63.6%)	5(50%)
**Treatment**			
Partial/Radical nephrectomy	2/19 (9.5%/90.5%)	1/10 (9.1%/90.9%)	1/9(10%/90%)
Nivolumab	21 (100%)	11 (100%)	10 (100%)
Sunitinib/Adjuvant Radiation	18/0 (85.8%/0%)	18/0 (85.8%/0%)	18/0 (85.8%/0%)
**RCC (B/L //U/L)**	1/20 (4.8%/95.2%)	1/10 (9.1%/90.9%)	0/10 (0%/100%)
**Clinical End point**			
Dead/Alive	3/18 (14.2%/85.8%)	1/10 (9.1%/90.9%)	2/8 (20%/80%)
Clinical complications (including Dead)/Stable	8/13 (38%/62%)	4/7 (36.3%/63.6%)	4/6 (40%/60%)
**Histology**			
P T1	3 (14.2%)	2 (18.2%)	1 (10%)
P T2	1 (4.8%)	0 (0%)	1 (10%)
PT3	17 (80.9%)	9 (81.8%)	8 (80%)
**Gross Tumor Volume**	305 cc (Median)		
**Histology**			
Clear cell	18 (85.8%)	8 (72.7%)	10 (100%)
Papillary	2 (9.5%)	2 (18.1%)	0 (0%)
MIT family	1 (4.7%)	1 (9.1%)	0 (0%)
**Sarcomatoid elements**			
Present	6 (28.5%)	2 (18.2%)	4 (40%)
Absent	15 (71.5%)	9 (81.8%)	6 (60%)
**Fuhrman’s Grade**			
2	5 (23.8%)	3 (27.2%)	2 (20%)
3	14 (66.7%)	8 (72.8%)	6 (60%)
4	2 (9.5%)	0 (0%)	2 (20%)
**Renal pelvis**			
Involved	7 (33.3%)	4 (36.3%)	3 (30%)
Not Involved	14 (66.7%)	7 (63.7%)	7 (70%)
**LVNI**			
Present	12 (57.1%)	8 (72.7%)	4 (40%)
Absent	9 (42.9%)	3 (27.2%)	6 (60%)
**Lymph nodes**			
Present	4 (19%)	3 (27.2%)	1 (10%)
Absent	17 (81%)	8 (72.8%)	9 (90%)
**Gerotas fascia, ureter, and renal vessels**			
Involved	0 (0%)	0 (0%)	0 (0%)
Not involved	21 (100%)	11 (100%)	10 (100%)

**Table 2:** Median of mean tumor sizes and organ-specific responses to CPI in HCC.

	Liver (n = 10)	Lung (n = 26)	Brain (n = 5)	Lymph Node (n = 20)	Soft-Tissue (n = 7)	Adrenals (n = 4)	Peritoneum (n = 4)
**Median Size Baseline (in mm)**	20	20	12	13	30	19	15.5
**Median Size LFU (in mm)**	19	20	25	12	40	25	20
**PR**	1	5	1	7	0	1	1
**SD**	4	3	0	5	1	1	0
**IUPD**	2	5	3	3	1	1	2
**ICPD**	3	12	1	5	5	1	1
**Objective Response (%)**	10	19	20	35	0	25	25

## Data Availability

All data generated and analyzed during this study are included in this published article.

## Abbreviations

CR	Complete remission
CT	Computed Tomography
RCC	Renal cell carcinoma
ORR	Objective response rate
OSRR	Organ-specific response rate
PD	Progressive disease
PD-L1	Programmed death-ligand 1
CPI	Check point inhibitors
LVNI	Lymphovascular neural invasion
ICPD	Immune-confirmed Progression
IUPD	Immune-unconfirmed Progression
LFU	Last follow up
